# Dual blockage of both PD-L1 and CD47 enhances immunotherapy against circulating tumor cells

**DOI:** 10.1038/s41598-019-40241-1

**Published:** 2019-03-14

**Authors:** Shu Lian, Ruizhi Xie, Yuying Ye, Yusheng Lu, Yunlong Cheng, Xiaodong Xie, Shuhui Li, Lee Jia

**Affiliations:** 1 Cancer Metastasis Alert and Prevention Center, College of Chemistry; Fujian Provincial Key Laboratory of Cancer Metastasis Chemoprevention and Chemotherapy, Fuzhou University, Fuzhou, China; 2 Fujian Provincial People’s Hospital Affiliated to Fujian University of Traditional Chinese Medicine, Fuzhou, 350004 China; 3 Marine Drug R&D Center, Institute of Oceangraphy Minjiang University, Fuzhou, 350108 China

## Abstract

Carcinoma metastasis is triggered by a subpopulation of circulating tumor cells (CTCs). And single immune checkpoint therapy is not good enough to inhibit CTC-induced metastasis. Here, we demonstrate that simultaneously blocking CD274 (programmed death ligand 1, PD-L1 or B7-H1) and CD47 checkpoints which were respectively signal of “don’t find me” and “don’t eat me” on CTCs by corresponding antibodies could enhance the inhibition tumor growth than single CD274 or CD47 antibody alone. *In vitro* flow cytometry data proved that CD47 and CD274 were overexpressed on the tested mouse tumor cell lines. The antibodies could effectively block the expressions of CD47 and CD274 on the cell surface and stably attached to tumor cell surface for several hours. The simultaneous blockade on both CD47 and CD274 checkpoints inhibited tumor growth and CTCs metastasis more potently than a single antibody inhibition or blank control on 4T1 tumor mouse model *in vivo*. Our results demonstrated that simultaneous dual targeting immune checkpoints, i.e., CD47 and CD274, by using specific antibodies may be more effective as an immunotherapeutics on CTCs than a CD47 or CD274 alone.

## Introduction

Tumor metastasis is a complex process caused by the spread, seeding and implantation of malignant cancer cells of primary tumor^1–3^, which is the leading cause of cancer-related death^4^, resulting in 90% cancer patients death^5^. Once CTCs escaped from immune system, cancer metastasis could be initiated by such a subpopulation of escaping CTCs in blood. PD-L1 (or CD274) protein has emerged as one such candidate biomarker capable of predicting whether patients are more likely to respond to immunotherapy^6^. In contrast to most traditional medicine treatment, immunotherapy may attain non-toxicity, effective and reliable clinical efficacy^7^. Not only that, a number of recent studies about antibody-mediated blockade of PD-L1 on various cancers including non-small-cell lung cancer, melanoma, and renal-cell cancer had been reported^8–10^.

However, the relative treatment of PD-L1 is only suitable for high-PD-L1 expression cancer patients but it may not fit for low-PD-L1 expression cancer patients. Interestingly, CD47 is also known as a key immune checkpoint which is highly expressed on tumor cells, making tumor cells resistant to host immune surveillance^11^. By blocking the CD47/SIPR-α axis using monoclonal antibody also has great potential and advantage in immunotherapeutic strategies for acute myeloid leukemia^12^, breast cancer^13^, and small cell lung cancer^14^. In general, PD-L1 is a critical “don’t find me” signal to the adaptive immune system^15–17^, whereas CD47 is a critical “don’t eat me” signal to the innate immune system as well as a regulator of the adaptive immune response^18,19^. Considering that CTCs usually simultaneously expresses more than one immune checkpoint^20^, thus we hypothesized that blocking multiple immune checkpoints such CD47 and CD274 can simultaneously restrain more biomarkers in CTCs with stronger immune recognition avidity.

To confirm the hypothesis and realize more effective treatment and inhibition immune evasion of CTCs, we selected mice tumor 4T1 cells as a CTC model, and blocked two immune checkpoints: CD47 and CD274. This may be a new and good method for treatment of tumor metastasis, and at the same time it could be a solution to the limitation of single antibody limitation, fewer checkpoints and poor targeting.

## Results

**Figure 1:**
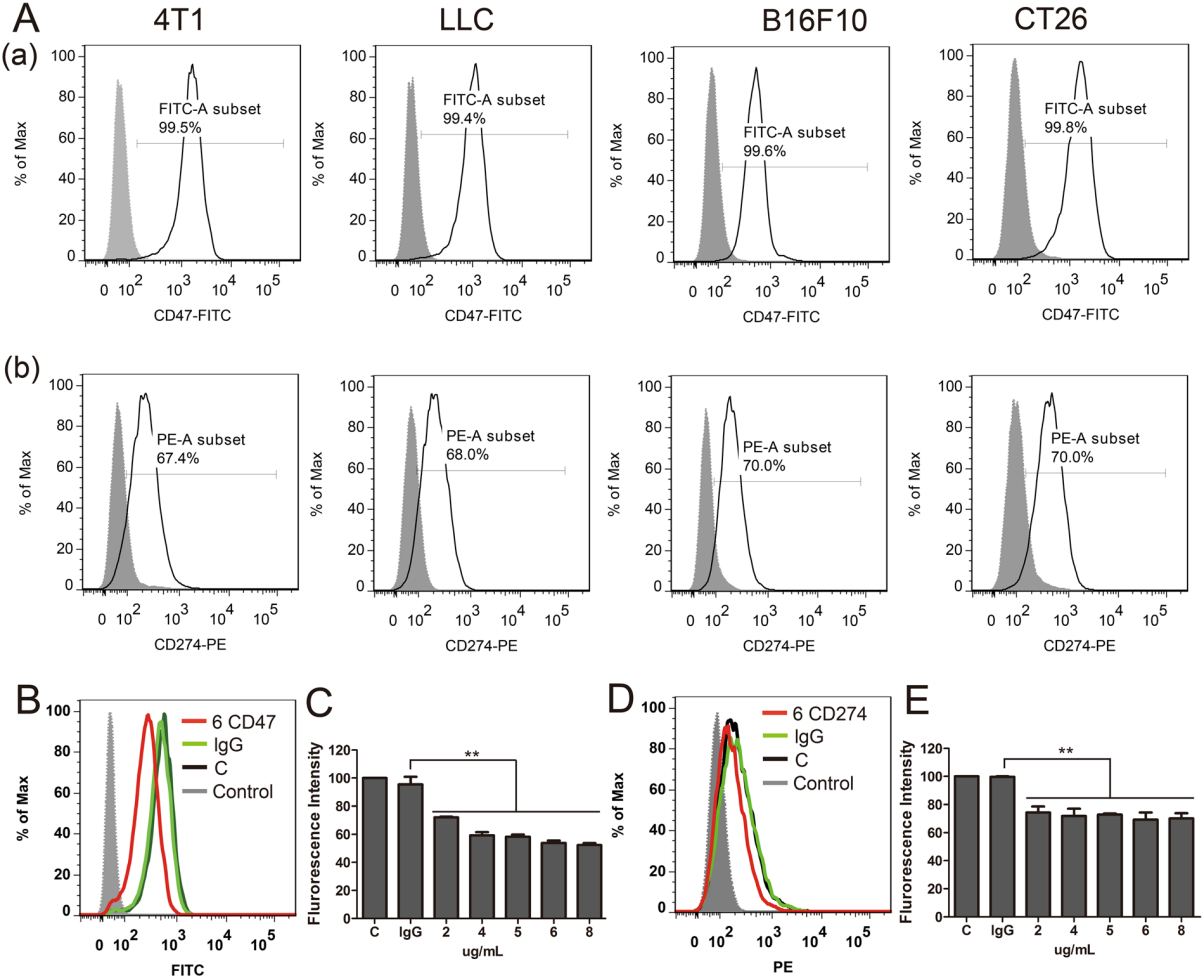
(**A**) Flow cytometric analysis of CD47 and CD274 expression of four mouse cancer cells (4T1, LCC, B16F10 and CT26). (**B**,**E**) Flow cytometric analysis the CD47 and CD274 proteins expression on 4T1 cells treated by different antibodies treatment. Control, isolated control; C, cells alone; lg G, the 4T1 cells (10^5^) were incubated with 6 μg anti-lg G; 6 CD47/CD274, the 4T1 cells (10^5^) were incubated with 6 μg anti-CD47/CD274. Error bars show mean ± SEM (**P < 0.01).

First, we determined expression of CD47 and CD274 in mouse cancer cells. As shown in Fig. 1A, mouse cancer cells −4T1, LCC, B16F10 and CT26- had CD47^+^ cells with high expression rates of 99.5%, 99.4%, 99.6%, 99.8%, respectively, in comparison with the isotype control. Similarly, the four mouse tumor cells had CD274^+^ cells with expression rates of 67.4%, 68.0%, 70.0% and 70.0%, respectively, comparison to isotype control. There results validated that CD47 and CD274 were highly expressed in a series of mouse cancer cells.

To verify the inhibition effect of anti-mouse CD47 and anti-mouse CD274, after incubation with purified anti-mouse antibodies, the cells were dyed with the fluorescence antibodies and determined by flow cytometric. As shown in Fig. 1B–E, the expression of CD47 and CD274 was respectively reduced to 51.2 ± 0.6% and 70.6 ± 1.2% in 30 min at the antibody concentration of 6 μg/1 × 10^5^ cells, and the blockade effect increased with increasing concentration.

**Figure 2:**
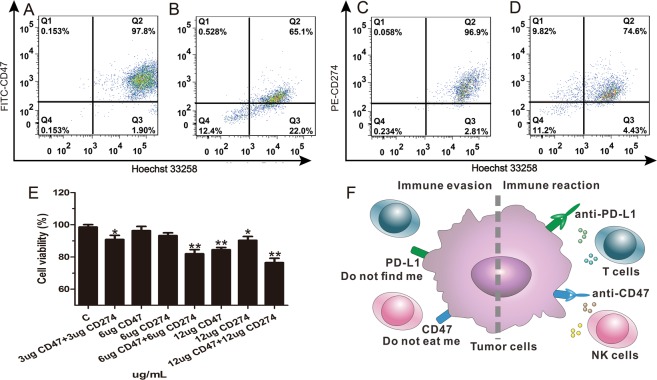
**(A–D)** Flow cytometric analysis showed percentages of FITC^+^ CD47 antibody bind to Hoechst 33258^+^ 4T1 cells for 0 h (**A**) or 3 h (**B**) and PE^+^ CD274 antibody bind to Hoechst 33258^+^ 4T1 cells in whole blood for 0 h (**C**) or 3 h (**D**). (**E**) Cell viability of 4T1 cells incubated with mouse blood monocyte. (**F**) Schematic diagram depicts that dual inhibition on both CD47 and PD-L1 changes immune evasion to immune activation by T cells and NK cells.

The binding abilities between antibodies and antigens on cells were also detected by flow cytometer. The data (Fig. 2A–D) were collected regarding Hoechst 33258-positive, FITC-positive/PE-positive, and double-positive events, the last events interpreted as binding ability of antibodies and cells. Flow cytometry analysis exhibited that the binding functionality between anti-CD47/anti-CD274 and 4T1 cells remained in a high level after 3 h incubation in whole blood. The binding percentage between cells and antibodies maintained at 65.1% of CD47^+^ and 74.6% of CD274^+^. The results indicated that antibodies can bind stably to antigens on cells, at ratio of cells to RBCs was equal to 1:10^3^. To detect synergistic effect of two antibodies, 4T1 cells were co-incubation with mice blood monocyte. As shown in Fig. 2E, the 4T1 cell viability of 3 μg/mL anti-CD47 and 3 μg/mL anti-CD274 group was significant decrease compared to 6 μg/mL anti-CD47 group or 6 μg/mL anti-CD274 group. This indicated that the antibodies of CD47 and CD274 had synergistic effects in immune therapy. Besides, the Fig. 2F was schematic diagram.

**Figure 3:**
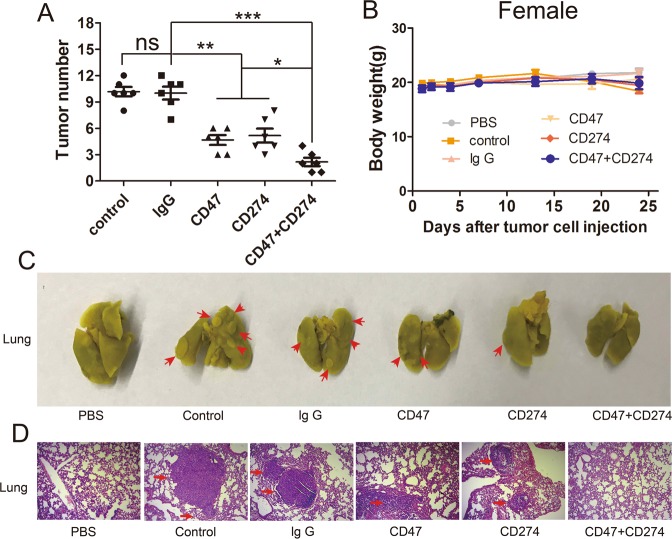
(**A**) Tumor nodules in the lungs of the treated mice developed within 24 days after injection of the 4T1 cells. (**B**) The body weight variation of 4T1 tumor-model mice during the treatments. (**C**) The gross appearance of tumor nodules of 4T1 (shown in arrows) in lungs after fixation with Bouin’s solution for 24 h. (**D**) Histological staining of tumor-bearing lung tissue section with H&E after different treatment, amplification × 100. PBS. PBS only, Control. Tumors only, lg G. Tumors with control lg G, CD47. Tumors with anti-CD47, CD274. Tumors with anti-CD274, CD47 + CD274. Tumors with anti-CD47 and anti-CD274.

To evaluate whether synergy blocking of CD47 and CD274 on cancer cells could be effectively against CTCs in the lungs, we employed a well-established CTCs 4T1 model, which spontaneously developed lung metastasis. The 4T1 cells were blocked with CD47 antibody or/and CD274 antibody before injection. After 24 days treatment, the lungs of mice were harvested and tumor nodules were calculated after Bouin’s fixation. The tumor nodules of the control or lg G antibody group indicated the successful establishment of 4T1 lung metastasis in Balb/c model. In the anti-CD47 or anti-CD274 treated group, tumor nodules were reduced in the lungs (Fig. 3). Surprisingly, there were no obvious tumor nodules in the anti-CD47 or anti-CD274 group compared with other control groups (Fig. 3A). Treated mice did not lose weight during treatment (Fig. 3B), which suggested no systemic toxicity during the treatment. Lung metastasis was further assessed by H&E staining after paraffin section (Fig. 3D). H&E assay confirmed that apoptosis and necrosis areas were the greatest in the CD47 and CD274 group as the arrows pointed. The control-treated or lg G-treated group H&E examination on lung tissue showed compact tumor cells with intact structure. Meanwhile, our results indicated that there was no obvious tumor cells in the anti-CD47 and anti-CD274 group, suggesting that synergy CD47 antibody and CD274 antibody could effectively prevent CTCs from forming metastases in the lungs. The sections of heart, liver, spleen and kidney were also tested by H&E staining, but there were no organ damaged appeared (Fig. S1). The examinations showed that there was not any significant signs of toxicity in other organ by anti-CD47 or anti-CD274.

**Figure 4:**
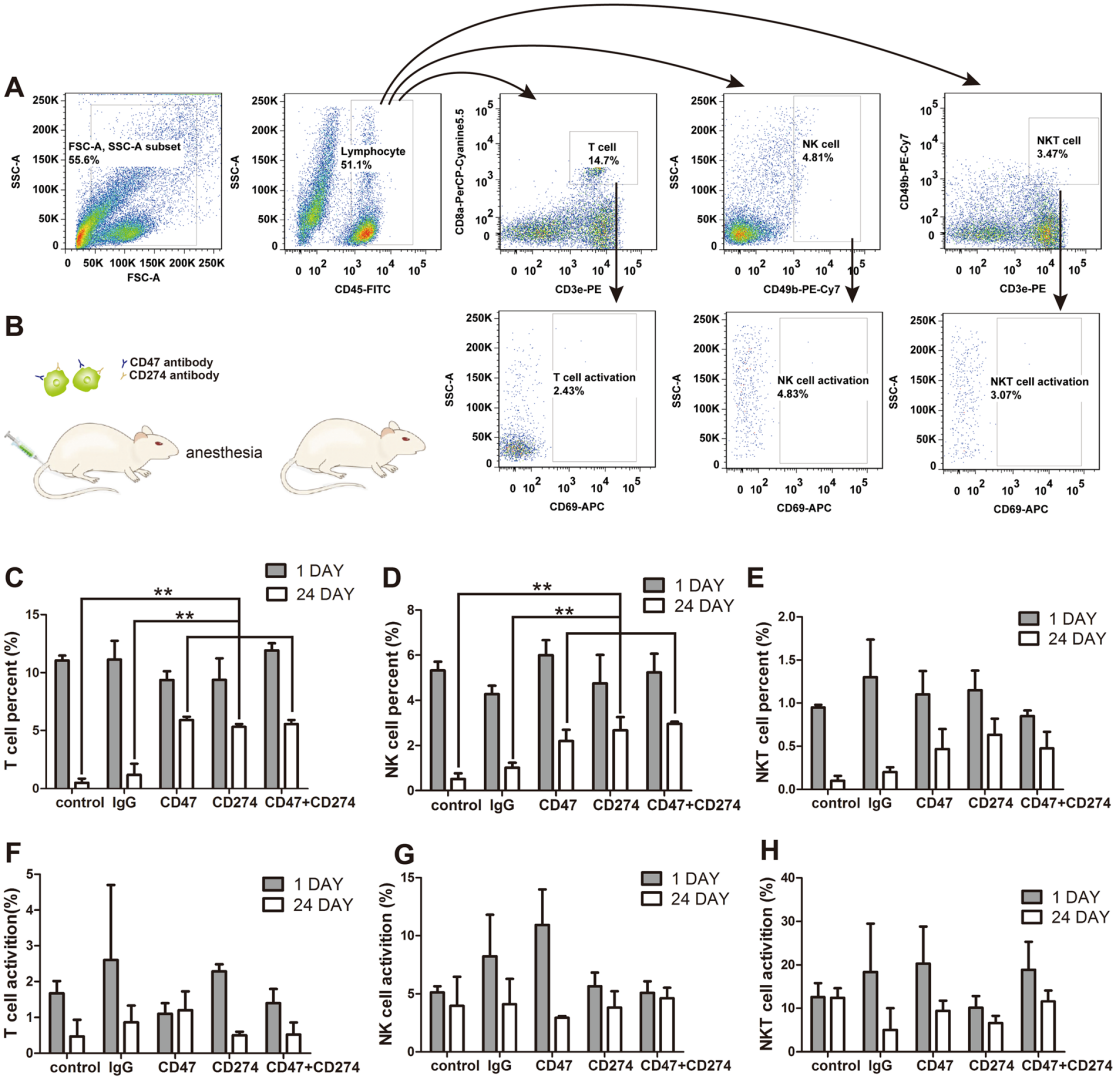
*In vivo* anti-tumor efficacy of two antibody blockade both CD47 antibody and CD274 antibody in 4T1 tumor cells. (**A**) Flow cytometry sorting graphs showing how to distinguish unwanted noise from gated. (**B**) Schema for two antibodies blockade in immunotherapy. (**C–E**) The percent of T cells, NK cells and NKT cells in leukocyte since the start of the injection (*P < 0.05, **P < 0.01 and ***P < 0.001). (**F–H**) The percentage activation of T cells, NK cells and NKT cells after treatment.

In blood the changes of T cells, NK cells and NKT cells on 1 day and 24 day were detected by flow cytometry. Blood samples were collected from mice eyes and processing step was followed by pre-described 2.8 method. The relative flow cytometer analysis process was shown in Fig. 4A. The mice were anesthetised by ether during blood collection period, and Fig. 4B was the schema for experiment. In a syngeneic model of lung metastatic cancer, with the blockade of CD47 or/and CD274 the percentage of T cells (Fig. 4C) and NK cells (Fig. 4D) resulted in a significant enhance the immune cells’ cytotoxic ability. This means that as long as one of CD47 or CD274 is blocked, immune cells can maintain highly activity.

There was a slight increase in NKT cells compared with the blank group but there was no significant difference between other five groups (Fig. 4E). Meanwhile, the activation percentages of T cells, NK cells and NKT cells were no obvious difference among control group, lg G group, CD47 group, CD274 group and CD47 and CD274 group (Fig. 4(F–H)**)**.

## Discussion

The immune system responds to many different receptor–ligand pairs, which may help activate immune cells. However, only blocking one checkpoint may not satisfy treating the circulating tumor cells. Targeting CD47 or CD274 (PD-L1) is very commonly used in immune checkpoint therapy.

We confirmed THE expression of CD47 and CD274 in a series of mouse cancer cells (Fig. 1A). The high-expression of CD47 and CD274 on cancer surface may be the reason why circulating tumor cells could escape from the immune system. Anti-CD47 and anti-CD274 could blockade CD47 and CD274 expression on 4T1 cancer cells (Fig. 1B–E). In whole blood the binding function between antibody and 4T1 cells remained high levels for 3 hours (Fig. 2A–D). After incubation with mice blood monocyte cells, synergistic anti-CD47 and anti-CD274 group had obviously toxic effect (Fig. 2E). Our results demonstrated that synergism blockade CD47 and CD274 antibody in cancer can also inhibit tumor growth and CTCs metastasis *in vivo* (Fig. 3), which may block CD47 binding to SIPR-α and PD-L1 binding to PD-1. Blocking PD-L1 on tumor is generally considered to enhance the activity of effector T cells (Fig. 4C) in the tumour micro-environment, and it also enhanced NK cell (Fig. 4D) activity and may enhance production through indirectly or direct effects on PD-1^+^ B cells^21^. And there being ample evidences proved that CD8^+^ cytotoxic T cells and natural killer (NK) cells were involved in the elimination of some viruses, in graft rejection^22^, in anti-tumour immune response, in immunopathology and some autoimmune diseases^23^. Besides, CD47 could enhance antitumor inflammation and T-cell recruitment in a DC-manner^24^. Thus, both CD47 and CD274 proteins contribute to the tumor micro-environment by affecting T cell activation and angiogenesis^16,25–29^, and the results confirmed that the percentage of T cells and NK cells were increased with the blockade of CD47 or/and CD274. Through coordination blockade the expression of CD47 and CD274 in tumor, the immune system can maintain the high quality of T cells and NK cells *in vivo* and prevent the immune escape of CTCs.

Our data demonstrated that the synergy anti-CD47 and anti-CD274 showed the least number of tumorigenesis, and the inhibition cancer metastasis effect was more obvious than antibody alone. Blocking both CD47 and CD274 may be a good method for treatment of tumor metastasis, and at the same time this could be a solution to the limitations of single antibody limitation, fewer checkpoints and poor targeting. In our study 4T1 cells were used as a CTC model. It has been confirmed that combination CD47 and CD274 is an excellent method for cancer therapy. We hope that our study, using dual-immune marker checkpoints, i.e., CD47 and CD274, may be useful to better treat CTCs and be inspiring of different treatment method.

## Methods

### Ethics statement

All animal experiments were approved by the Institutional Animal Care and Use Committee of Fuzhou University and operated following the NSFC regulations concerning the care and use of experimental animals. Thirty-six female Balb/c mice (about 20 g weight, 4–6 weeks old) were obtained from Fuzhou Wushi Animal Center.

### Antibodies and chemicals

Anti-mouse CD274 (PD-L1 or B7-H1)-PE, anti-mouse CD47-FITC, anti-mouse CD47 purified, anti-mouse CD274 purified, anti-mouse lg G purified, anti-mouse CD45-FITC, anti-mouse CD3e-PE, anti-mouse CD8a-PerCP-Cyanine5.5, anti-mouse CD49b (Integrin alpha 2)-PE-Cy7 and anti-mouse CD69-APC were obtained from eBioscience. Red cell lysate was purchased from Ding Guo biotechnology Co., Ltd. Ficoll-Isopaque was purchased from TBD biotechnology Co., Ltd.

### Cell culture

The 4T1, B16F10, LLC and CT26 cells were obtained from Cell Bank of Chinese Academy. Cells were cultured in 1640 medium (Sigma) supplemented with 10% fatal bovine serum (FBS, obtained from PAN), and maintained at 37 °C in a humidified atmosphere of 5% CO_2_. 4T1, B16F10, LLC and CT26 cells were respectively stained with anti-mouse CD47 (FITC-labeled) and anti-mouse CD274 (PE-labeled) antibody at 4 °C for 30 minutes in the dark. Background staining was performed in the same way with isotype-matched control. After staining, cells were washed with 1% FBS-PBS and resuspended in 500 μL of PBS. Flow cytometric analysis was carried out on the BD FACSAriaIII (BD Biosciences), and the obtained data were analyzed with FlowJo software.

### The inhibition effect

The 4T1 cells were cultured in a 6-well plates (1 × 10^5^ cells/well). The cells in each well were respectively blocked with 2 μg, 4 μg, 5 μg, 6 μg and 8 μg anti-CD47 and incubated in the dark at 4 °C for 30 minutes, then the cells were washed twice with PBS. The concentration of antibody CD274 and lgG was the same as that of the antibody CD47. After that, the blocked cells were incubated with anti-mouse CD47 (FITC-labeled) and anti-mouse CD274 (PE-labeled) antibody in the dark for another 30 min at 4 °C. After washed by PBS twice, the FITC or PE intensity fluorescence of cells were collected by flow cytometric.

### Antibodies-to-cell binding stability

The 1 × 10^5^ 4T1 cells/each were harvested with 0.25% trypsin and stained by Hoechst 33258 at 37 °C for 20 min. The cells were washed with 1% FBS-contained PBS twice and the stained cells were incubated with 6 μg anti-mouse CD47 (FITC-labeled) or 6 μg anti-mouse CD274 (PE-labeled) antibody in the dark for another 30 min at 4 °C. And then 4T1 cells were spiked into whole blood and swung 200 g in shaking incubation for 0 h or 3 h, the ratio of cells to RBCs is 1:10^3^. The double-positive fluorescence 4T1 cells were detected by flow cytometer.

### *In vitro* cytotoxicity studies

The cytotoxicity of monocyte for the CD47 or CD274-blocking cells *in vitro* was determined with MTT assay. The 4T1 cells were seeded into 96-well plates (1 × 10^4^ cells/well). Twenty four hours later, the cells were treated with anti-CD47 or/and anti-CD274 for 1 h at concentration of 3, 6 and 12 μg/mL. Mice blood samples were collected from the eyes of mice which injected with 4T1 cells in advance. Monocyte were isolated from Ficoll-Isopaque density centrifugation. The monocyte were seeded in each wells and cultured with the CD47 and/or CD274-blocking 4T1 cells at ratio of 10:1. Twenty four hours later, MTT solution (5 mg/mL) was added. The results were measured with infinite M200 Pro microplate reader (Tecan, Switzerland). Cell viability (%) = OD of test group/OD of control group × 100% (Blank 4T1 cells with monocyte were used as a control group).

### Mouse model

Female Balb/c mice were divided into six groups (n = 6 mice/ group) randomly. Group 1 was intravenously injected with saline, group 2 was injected with 100 μL PBS containing 1 × 10^5^ 4T1 cells to each mouse. Group 3, group 4 and group 5 were intravenously injected with 100 μL PBS containing 1 × 10^5^ 4T1 cells which were incubated with anti-lgG, anti-CD47 or anti-CD274 respectively, at equivalent 0.3 mg/kg dose and were centrifuged to remove free antibody. Group 6 was intravenously injected with 100 μL PBS containing 1 × 10^5^ 4T1 cells which were incubated with anti-CD47 and anti-CD274 simultaneously, at equivalent 0.3 mg/kg dose and centrifuged to remove free antibody.

### Mouse blood treatment

Mice blood samples were collected from the mice eyes with the capillaries. Fifty microliter of mice blood was collected from each mice and stored in 1.5 mL EP tube containing ethylenediaminetetraacetic acid. Cell pellets were incubated with anti-CD19a, anti-CD3, anti-CD45, anti-CD8a, anti-CD69 and anti-CD49b for 30 minutes at 4 °C in the dark. Red blood cell lysis buffer (10-fold volume) was then added to the blood samples to remove the red blood cells (RBCs). Cell suspensions were pooled and centrifuged 300 g for 5 min at room temperature. After that, cells were washed with 1% FBS-contained PBS for twice before determined. Flow cytometric analysis data were collected on the BD FACSAria III (BD Biosciences).

### Statistical analysis

Graphpad Prism 5.0 (Graphpad software, San Diego, CA) was used for all statistical analyses. The mean ± S.E.M. was determined for each treatment group in the individual experiments, and the standard t-test was used to determine the distinction between treatment groups and control group(s). P-values < 0.05 were significant. Statistical analyses were performed with the SPSS statistical software package.

## Supplementary information

supporting information
