# Supplementary material for: Dual blockage of both PD-L1 and CD47 enhances immunotherapy against circulating tumor cells
Source: Sci Rep. 2019 Mar 14;9:4532. doi: 10.1038/s41598-019-40241-1 (PMC6418176; doi:10.1038/s41598-019-40241-1)
Supplement: Supplementary file 1 — supporting information [file 41598_2019_40241_MOESM1_ESM.docx]

**Dual blockage of both PD-L1 and CD47 enhances immunotherapy against circulating tumor cells**

Shu Lian^1#^, Ruizhi Xie^1#^, Yuying Ye^2^, Yusheng Lu^1^, Yunlong Cheng^1^, Xiaodong Xie^1^, Shuhui Li^1^, Lee Jia^1,3^ *

^1^ Cancer Metastasis Alert and Prevention Center, College of Chemistry; Fujian Provincial Key Laboratory of Cancer Metastasis Chemoprevention and Chemotherapy, Fuzhou University, Fuzhou, China.

^2^ Fujian Provincial People's Hospital Affiliated to Fujian University of Traditional Chinese Medicine, Fuzhou, 350004, China.

^3^ Marine Drug R&D Center, Institute of Oceangraphy Minjiang University, Fuzhou, 350108, China

* Corresponding author: Lee Jia; Sunlight Building, 6FL; Science Park, Xueyuan Road, University Town; Cancer Metastasis Alert and Prevention Center, Fuzhou University, Fuzhou, Fujian 350116, China. Email addresses: cmapcjia1234@163.com or [pharmlink@gmail.com](mailto:pharmlink@gmail.com).

**^#^** These authors equally contributed to the work.

**Running title: Dual-immune checkpoints in PD-L1 and CD47**


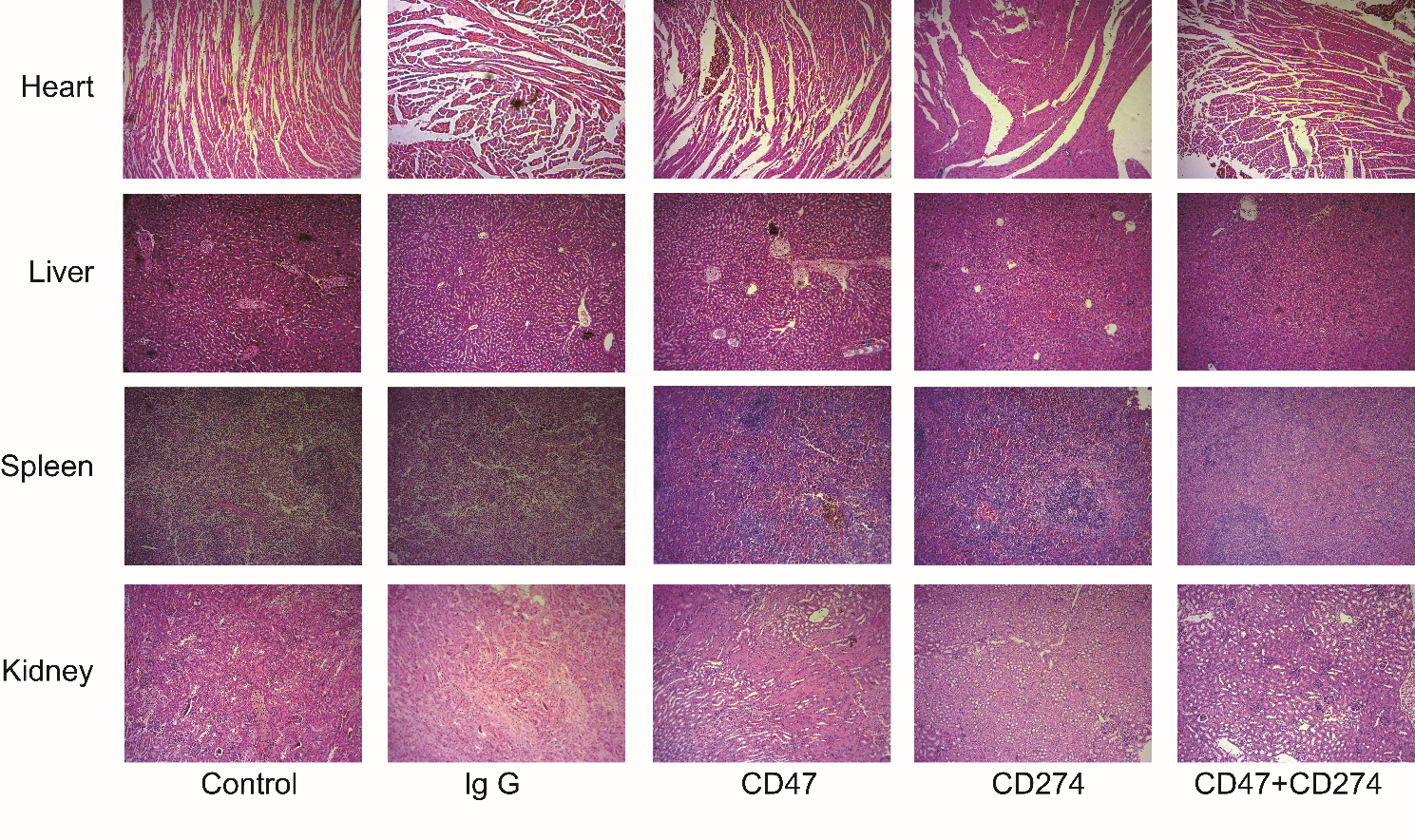


**Supporting Fig.1.** In vivo toxicity assays in normal mice. H&E of different organs of normal mice with different treatments. Control. Tumors only, lg G. Tumors with control lg G, CD47. Tumors with anti-CD47, CD274. Tumors with anti-CD274, CD47+CD274. Tumors with anti-CD47 and anti-CD274. Data being representative with at least 3 mice.
